# “Working Deeply With the Community”: Nurse‐Community Partnership Processes for Climate Justice

**DOI:** 10.1111/phn.70044

**Published:** 2025-11-17

**Authors:** Jessica LeClair, Kelly Krainak, Linsey Steege, Susan Zahner

**Affiliations:** ^1^ School of Nursing University of Wisconsin–Madison Madison Wisconsin USA

**Keywords:** authentic partnerships, climate justice, community‐based organizations, nurse‐community partnerships, planetary health, public health nurses

## Abstract

**Objective:**

To explore how public health nurses (PHNs) and community‐based organizations (CBOs) collaborate to advance climate justice, including their partnership processes, perceived facilitators and barriers, and the values that shape their relational work.

**Design:**

A qualitative descriptive study using photovoice and semi‐structured interviews, analyzed through reflexive thematic analysis.

**Sample:**

Eight PHNs and five CBO representatives from six US states participated, forming five PHN‐CBO dyads and three solo PHNs.

**Measurements:**

Data were collected from August 2022 to February 2023. Participants took photographs to guide discussion during joint photovoice sessions and participated in individual semi‐structured interviews. Transcripts were coded deductively, using the Authentic Partnerships Model, and inductively to identify emergent themes.

**Results:**

Participants described partnership processes that were grounded in trust, care, and a shared purpose. They emphasized the importance of power‐sharing, capacity building, and mutual respect in sustaining long‐term collaboration. Geographic distance and institutional constraints emerged as barriers to advocacy and connection. Personal values, community ties, and a desire for relational accountability in climate justice work shaped pathways into partnership.

**Conclusions:**

Findings expand current models of partnership and suggest that PHNs play a critical role in advancing climate justice through reflective, values‐driven collaboration with communities.

## 1 Background

Racialized and low‐income communities experience significant public health inequities due to overexposure to climate change, constituting climate injustice (Benz and Burney 2021; Bullard 1994; Hayden et al. 2023; Rockstrom et al. 2023). To address these inequities, public health departments are called to “partner across multiple sectors and leverage data and resources to address social, environmental, and economic conditions that affect health and health equity” (DeSalvo et al. 2017). A growing body of research underscores that such efforts are more effective in partnership with community‐based organizations (CBOs), which are grassroots groups led by community residents that address health, equity, and environmental justice (Baptista et al. 2023; Buse and Patrick 2020; Forman et al. 2016; Lemon et al. 2023; National Community‐Based Organization Network 2004).

However, many public health departments report limited capacity to build authentic, sustained partnerships with CBOs to prevent and address climate‐related health inequities (Lemon et al. 2023). Public health nurses (PHNs), the largest segment of the public health workforce, are well‐positioned to bridge this gap (Bekemeier et al. 2025). Working at the intersection of clinical, public health, and community practice, PHNs promote health equity and often serve as liaisons to CBOs (ANA 2022; Bekemeier et al. 2025; Kuehnert et al. 2022).

PHNs have long functioned as relational bridges between health systems and communities, engaging in partnership roles that extend beyond direct care. Studies have documented the role of nurse‐community partnerships in advancing community capacity for health promotion (Kang 1995) and guiding community‐led change (Anderson et al. 2002). In recent years, PHNs have been recognized for their contributions to community resilience and social infrastructure, especially amid public health stressors (Duva et al. 2022; Williams et al. 2018). Their evolving role in community health promotion has included coalition building, participatory interventions, and collaboration with grassroots organizations (Kulbok et al. 2012). In collaborative public health efforts such as the co‐development of COVID‐19 vaccine uptake programs, health actors have partnered directly with communities to design interventions responsive to local needs (Enlow et al. 2024). These examples show that PHNs already bring strengths in trust‐building, mutual accountability, and contextual knowledge to public health practice. Drawing on these established approaches, climate justice work represents a natural and needed extension of public health nursing partnerships. The significance of nurse‐community partnerships is well documented in the nursing literature on environmental justice (Amiri and Zhao 2019; Cantu et al. 2016; Kerr et al. 2021; LeClair, Kunkul, et al. 2024; Postma 2008). These partnerships are also endorsed by the *Code of Ethics for Nurses*, which states, “To transform unjust structures and directly address social and structural determinants of health, nurses must partner directly with communities of interest to advocate for community‐based organizations.” (American Nurses Association 2025, 34).

Within nursing, climate justice centers the knowledge, experiences, and leadership of communities disproportionately burdened by climate change and pollution (LeClair et al. 2022). Yet, limited published research exists on how PHNs and CBOs form and sustain partnerships for climate justice (LeClair, Watts, et al. 2021; Lilienfeld et al. 2018; Polivka and Chaudry 2018). To address that gap, this study asked the following research question: How do PHNs and CBOs collaborate to advance climate justice, including their partnership processes, perceived facilitators and barriers, and the values that shape their relational work?

## 2 Methods

### 2.1 Design

This qualitative descriptive study examined the nurse‐community partnership process for climate justice using two data collection methods: photovoice and semi‐structured interviews. Both methods aimed to describe the lived experiences of PHNs and CBO partners as they collaborated on climate justice initiatives.

#### 2.1.1 Ethical Considerations

The University of Wisconsin‐Madison Minimal Risk Research IRB determined that the study met the criteria for exemption (ID: 2022‐0771). All participants received a Research Information and Consent document outlining the study's purpose and were given opportunities to ask questions during enrollment and throughout the study.

#### 2.1.2 Theoretical Framework

**FIGURE 1:**
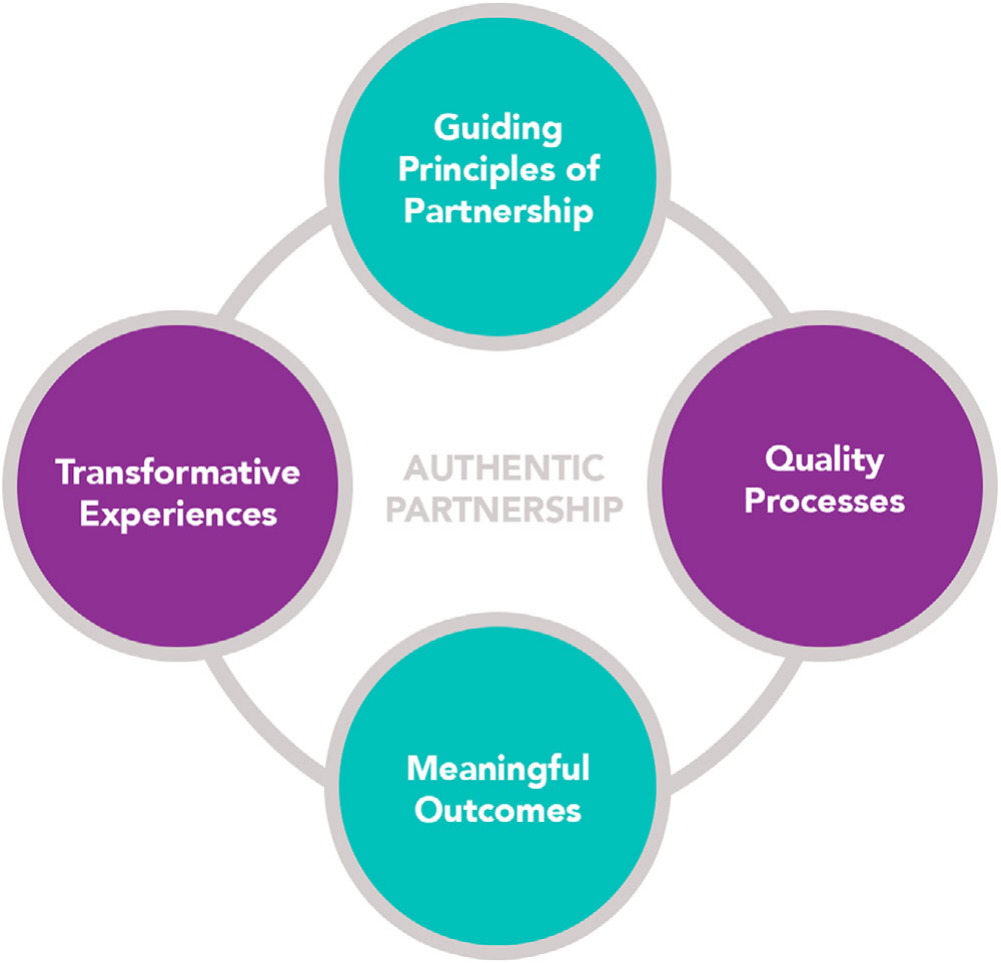
Authentic partnership model. [Colour figure can be viewed at wileyonlinelibrary.com]

The Authentic Partnerships Model, developed by the American Public Health Association's CBO Caucus, informed this study (National Community‐Based Organization Network 2004). This model, developed to strengthen collaborations between academia and CBOs, identifies four key elements of effective partnership: (1) Guiding Principles of Partnership, (2) Quality Processes, (3) Transformative Experience(s), and (4) Meaningful Outcomes (Figure 1).

In keeping with a reflexive thematic analysis approach (Braun and Clarke 2019), this model served as a conceptual lens to help interpret the data, particularly the elements of Guiding Principles, Quality Processes, and Transformative Experiences, which aligned with the study's aim. These elements emphasized shared purpose, mutual learning, and trust‐based relationship building. While these concepts helped orient the analysis, inductive coding was also employed to ensure that interpretations remained grounded in the participants’ narratives and themes that extended beyond the framework. This approach reflects the reflexive nature of the analysis, which acknowledges the role of the researcher's positionality and evolving understanding in shaping the interpretation of the data (Braun and Clarke 2019).

### 2.2 Sample

Participants were recruited nationally by distributing a study description and screening survey through 130 organizations, including the Council of Public Health Nursing Organizations (*n* = 7), state‐level nursing organizations (representing 49 states and two territories), public health organizations (*n* = 54), and climate and health organizations (*n* = 18). Recruitment materials were shared via organizational email lists, newsletters, and websites. Snowball sampling was also used, with all 130 organizational contacts being invited in the recruitment material to forward study invitations or refer nurses directly to the research team.

#### 2.2.1 Inclusion and Exclusion Criteria

Nurses were eligible if they (1) were Registered Nurses, (2) identified through the survey as engaging in population‐focused nursing practice, and (3) had experience partnering with a CBO to advance climate justice in a community. CBO representatives were eligible if they (1) were identified by enrolled nurses as partners and (2) confirmed that they had worked with the nurse to advance climate justice. The nurse could participate alone for partial data collection if a CBO partner was unavailable.

Fifty‐two nurses completed the screening survey and met initial inclusion criteria, with eight more referred through snowball sampling. Of these, 31 did not respond to follow‐up, and 21 either declined, cited scheduling constraints, or were deemed ineligible. Eight PHNs and five CBO representatives enrolled, including five PHN‐CBO dyads and three individual PHNs. PHNs were employed in academic or nonprofit settings, and study participants were in Massachusetts, Michigan, New Jersey, Ohio, Washington, and Wisconsin.

All participants provided verbal consent during a Zoom enrollment session and subsequently signed an electronic consent form. Everyone who completed all study activities, including both photovoice and semi‐structured interviews, received a $150 incentive, based on available funding and IRB guidance.

### 2.3 Measures

Data were collected between August 2022 and February 2023 using a combination of methods, including photovoice sessions and semi‐structured interviews. These two methods were used to explore how PHNs and CBO representatives collaborate to promote climate justice.

#### 2.3.1 Photovoice

Photovoice was conducted with PHN‐CBO dyads using a modified version of the method developed by Wang and Burris (1997). Each participant was invited to take photographs representing their experiences with climate justice, then prioritize a selection of images for discussion. Dyads participated in joint interviews with the first author to analyze and reflect on their images, generating rich narratives about their work and shared context.

Although the photovoice sessions did not directly solicit information about partnership processes, participants frequently reflected on their collaboration. These data were therefore included in the current analysis of partnership processes. Full results from the photovoice analysis focused on climate justice experiences and strategies are published elsewhere (LeClair, Dudek, et al. 2024; LeClair et al. 2025).

#### 2.3.2 Semi‐Structured Interviews

In addition to the photovoice sessions, the first author conducted in‐depth interviews with each participant. These interviews focused on the participants’ experiences of partnering to promote climate justice. The semi‐structured interview guide included questions about how partnerships were formed, sustained, and navigated over time. The Critical Environmental Justice Nursing for Planetary Health Framework, the Social‐Ecological Model, and the Authentic Partnerships Model guided initial interview questions, which were then refined by the Community Advisors on Research Design and Strategies at the University of Wisconsin‐Madison (CCPH Board of Directors 2013; Golden and Wendel 2020; LeClair et al. 2021).

All interviews (photovoice and individual) were conducted virtually using a HIPAA‐compliant video platform, recorded, and transcribed verbatim. Photovoice sessions ranged from 60 to 90 min, and individual interviews lasted approximately 45–75 min. Graduate students assisted with transcription, and transcripts were reviewed for accuracy. Each participant was provided with their transcript and invited to make edits or corrections as part of a member‐checking process.

### 2.4 Analytic Strategy

We used reflexive thematic analysis to analyze 13 individual interviews (one per participant) and eight photovoice transcripts (one per PHN‐CBO dyad). This approach, rooted in Braun and Clarke's work (2019), acknowledges the researcher's subjectivity and reflexivity as central to knowledge production. Rather than applying a rigid coding framework, we used theory as a lens to enrich interpretation while remaining open to themes that emerged inductively from the data.

The analysis followed Braun and Clarke's six‐phase framework: (1) data familiarization, (2) initial coding, (3) theme development, (4) theme review, (5) theme naming and definition, and (6) report production (Braun and Clarke 2006). The first and second authors independently reviewed transcripts, listened to audio recordings, and wrote analytic memos. Coding was conducted using a hybrid approach. Deductive codes were sensitized by three elements of the Authentic Partnerships Model (Guiding Principles, Quality Processes, and Transformative Experiences) (CCPH Board of Directors 2013), while inductive coding allowed for identifying new and unexpected patterns, such as processes related to partnership formation.

Coded data were grouped into categories and iteratively refined into themes. We actively examined tensions between deductive and inductive findings to avoid forcing data into the theoretical model. We also triangulated across different data sources (individual interviews and photovoice discussions) and types of partnership (e.g., PHN‐CBO dyads vs. individual PHNs and CBO representatives).

Reflexivity was central to our analytic process. The first author maintained a reflexive journal throughout the study, documenting analytic decisions and her positionality as a public health nurse and researcher. Data saturation was achieved through iterative processes, including analytic memo writing after each interview, where the first author documented emerging insights and compared these across cases (Boeije 2002; Rubin and Rubin 2011). Peer debriefing with the second author created space for examining assumptions and multiple interpretations. Follow‐up interviews were not pursued, as initial interviews provided rich, conceptually complete accounts. We conducted cross‐case comparisons between PHN and CBO participants and among different types of partnerships to examine the consistency and depth of themes. During the coding and theme development phases, we observed that no new codes or concepts were emerging, and the final interviews reinforced existing patterns. Thematic coherence across data sources (e.g., individual interviews and photovoice sessions) and participant types provided confidence that the themes were fully developed. Based on these criteria, the research team determined that saturation had been reached, and additional data collection would likely yield minimal new insights.

## 3 Results

Eight PHNs and five CBO partners enrolled in the study. Many participants voluntarily shared aspects of their intersecting social identities during data collection, although demographic information was not formally collected. These included Indigenous, Black, White, women, men, and members of the 2SLGBTQ+ community. The participating PHNs either volunteered with or were employed by the CBO or academic institutions.

### 3.1 Themes

The findings correspond with the study's research question to identify processes that sustain nurse‐community partnerships for climate justice. Nurses and their community partners utilized processes aligned with the Authentic Partnerships Model (CCPH Board of Directors 2013). Participants also described how their partnerships were formed, which was not included in the Authentic Partnerships Model. The following two themes and six subthemes are given below and summarized in Table 1 in the following order: Theme 1: Processes to Sustain Nurse‐Community Partnerships; Subthemes: (1a) Prioritizing trust, respect, care, and commitment; (1b) Building upon identifying strengths and sharing power; (1c) Developing a shared purpose with evolving, transformative goals; (1d) Navigating geographic distance and uneven capacity. Theme 2: Pathways to Nurse‐Community Partnerships for Climate Justice. Subthemes: (2a) Having a strong internal foundation and (2b) Seeking connection with others.

**TABLE 1:** Nurse‐community partnership processes for climate justice.

Themes	Subthemes	Example excerpts
1. Processes to Sustain Nurse‐Community Partnerships	1a. Prioritizing trust, respect, care, and commitment. 1b. Building upon identified strengths and sharing power. 1c. Developing a shared purpose with evolving, transformative goals. 1d. Navigating geographic distance and uneven capacity.	1a: “It is important that a person be truthful and honest and respectful. Those are the priorities coming from our people, from our culture.” [CBO8]. 1b: “There's just this understanding… the reciprocity between us, that if I need something, I know that I can reach out and get support—and vice versa.” [PHN3]. 1c: “…that shared mission that really kind of unites… these little CBOs, these nonprofits—they'll get $1,000 and they'll do a lot with that $1,000… I'm so glad to be part of this.” [PHN7] 1d: “I wish I had been in [CITY] and had all of the local networks at my fingers.” [PHN4].
2. Pathways to Nurse‐Community Partnerships for Climate Justice	2a. Having a strong internal foundation. 2b. Seeking connection with others.	2a: “I want to enter environmental health nursing. I want to do something where I can stand up and speak out for injustices.” [PHN8]. 2b: “That was a huge moment—now I was able to go into human health and the injustice.” [CBO1].

Example excerpts are provided, beginning with results from our deductive analysis of partnership processes and ending with our inductive analysis of partnership formation. In the narratives below, the public health nurses are labeled “PHN,” and the community‐based organization representatives are labeled “CBO.” Theme names and quotes are given in italics.

#### 3.1.1 Theme 1: Processes to Sustain Nurse‐Community Partnerships

This theme and subthemes describe processes supporting maintaining and deepening nurse‐community partnerships for climate justice. These processes were deductively interpreted using the Authentic Partnerships Model (CCPH Board of Directors 2013), particularly the elements of Guiding Principles, Quality Processes, and Transformative Experiences.

Participants described how their partnerships were rooted in shared values and relational commitments beyond project goals or institutional roles. Rather than transactional collaborations, these partnerships were often described as evolving, multi‐dimensional relationships shaped by mutual accountability, emotional connection, and reciprocal support. The following subthemes illustrate how trust, care, power‐sharing, and purpose were enacted in practice, revealing what sustains partnerships and how these relational dynamics unfold over time in the context of climate justice work.

**1a. Prioritizing trust, respect, care, and commitment**. A foundational process sustaining nurse‐community partnerships was a deep commitment to trust, mutual respect, care, and relational accountability. Participants consistently emphasized that partnerships could not thrive without establishing trust, especially in communities with histories of marginalization or harm from institutional actors.

Several participants described trust not as a static attribute, but as something cultivated over time through actions that demonstrated presence, honesty, and respect. For example, one CBO explained, “It is important that a person be truthful and honest and respectful. Those are the priorities coming from our people, from our culture.” [CBO8]. This framing linked trust‐building to cultural values and historical resilience, particularly among Indigenous and Black communities.

The process of developing trust was described as layered and personal. A CBO reflected on how the PHN's consistent presence shifted perceptions over time: “Now the community's like, ‘Is [PHN] coming? [PHN] coming?’. because we've spent time showing that we're not here to do any harm.” [CBO4]. These reflections suggest that trust is experienced between the partners and extends to how community members perceive and engage with the partnership.

PHNs described how their commitment to the partnership extended beyond professional roles into their relationships with the community and their CBO counterparts. As one nurse explained, the work required showing up with care and empathy, not just clinical knowledge, “There's a lot of textures to the way you build a relationship that is meaningful…you demonstrate humanity, you care about each other's families.” [PHN1]. Several participants noted genuine care required acknowledging grief, joy, and shared humanity, not simply exchanging information. The depth of these relational bonds was described as essential for navigating challenges and sustaining momentum in climate justice work.

This theme emerged primarily through deductive coding, aligned with the Quality Processes and Guiding Principles of Partnership elements of the Authentic Partnerships Model (CCPH Board of Directors 2013). However, participants’ emphasis on familial and emotional dimensions of care deepened our understanding of how these values are enacted in practice, especially in communities historically harmed by extractive partnerships.

**1b. Building upon identified strengths and sharing power**. A core sustaining process described by participants was the intentional recognition and amplification of each partner's strengths. Rather than operating within hierarchical structures, participants emphasized the value of co‐leadership and reciprocal expertise. PHNs and CBO representatives spoke about “working in tandem,” each contributing unique knowledge grounded in public health, lived experience, cultural context, or organizing strategies. As one CBO reflected, “[PHN4] and I… work in tandem. It's a co‐partnership, you know. I know her strengths. She knows mine.” [CBO4].

This mutual recognition enabled each partner to act confidently within their domain while deferring to the other's expertise when necessary. Several PHNs described how they intentionally made space for their CBO partners to lead in community settings, recognizing the historical erasure of community knowledge in health system collaborations. One PHN explained, “There's just this understanding… the reciprocity between us, that if I need something, I know that I can reach out and get support—and vice versa.” [PHN3].

Partnerships also grew stronger when each partner advocated for the other's value in external settings. For example, a CBO recalled defending their PHN partner's expertise to others by saying, “You wanna talk about community? You ask me. Ask her about public health—she's gonna run circles around you with that. Us together, we kick ass and take names.” [CBO4].

This subtheme was interpreted deductively through the lens of the Authentic Partnerships Model's Quality Processes and Transformative Experiences elements, particularly the principles of mutual respect, shared credit, and capacity building. It also illuminated how power‐sharing was not symbolic but actively negotiated in daily practice, shaped by trust, humility, and a shared commitment to community well‐being.

**1c. Developing a shared purpose with evolving, transformative goals**. Participants described how sustained partnerships were anchored by a shared sense of purpose that often emerged through relational work and continued to evolve over time. Rather than beginning with fully aligned objectives, many partnerships grew into alignment through ongoing dialogue, mutual trust‐building, and shared experiences navigating climate justice work together. A common thread in participant narratives was the deep fulfillment nurses experienced when their professional expertise aligned with community‐driven missions. One PHN reflected, “…that shared mission that really kind of unites… these little CBOs, these nonprofits—they'll get $1,000 and they'll do a lot with that $1,000… I'm so glad to be part of this.” [PHN7].

The transformative nature of partnership was evident as goals shifted in response to emerging needs, local crises, and collective reflection. For example, participants shared how initial plans were adapted during the COVID‐19 pandemic to meet urgent community needs, offering protective equipment, clean drinking water, and health education in ways that deepened partnership trust and relevance. This adaptability required PHNs to embrace flexibility, particularly when institutional settings (e.g., academic) limited their ability to support the CBO's advocacy work. As one PHN explained, “I was told that I can present results, but I can't voice my support… There was a barrier between political activism and academia.” [PHN5].

Participants described how the process of co‐developing goals not only strengthened the partnership but also reshaped their own understanding of what climate justice required. This subtheme reflects the Authentic Partnerships Model's Transformative Experiences and Guiding Principles elements (CCPH Board of Directors 2013). It also illustrates the tensions that arise when individual and organizational values intersect, and the creative strategies partners use to navigate those tensions while sustaining momentum toward justice‐oriented goals.

**1d. Navigating geographic distance and uneven capacity**. Despite strong relational foundations, some partnerships encountered significant structural and geographic barriers that hindered their ability to sustain momentum and achieve shared goals. A recurring challenge was the geographic separation between PHNs and CBO partners, particularly when PHNs were based outside the community or in academic institutions far from the sites of community action. One PHN described how this distance limited her ability to support local connections for her partner, “I wish I had been in [CITY] and had all of the local networks at my fingers.” [PHN4]. This lack of embeddedness sometimes hindered the PHN's ability to link CBO partners to other local health resources or networks, especially in time‐sensitive climate‐related advocacy efforts. In contrast, community‐based partners brought deep relational ties and context‐specific expertise, often placing them at the frontline during crises like the COVID‐19 pandemic.

Several PHNs acknowledged asymmetries in access to institutional resources, noting that they could offer technical support or academic legitimacy, but not always the proximity or relational depth that CBO partners embodied. These reflections address a broader structural issue: the disparities in power, proximity, and institutional constraints that influence who is allowed to advocate or lead.

This subtheme was interpreted inductively, expanding beyond the Authentic Partnerships Model to name contextual limitations that influenced the ability to fully enact shared values like mutual support and equitable capacity‐building. While not dissolving the partnership, these constraints required ongoing negotiation and creativity to maintain relational integrity.

#### 3.1.2 Theme 2: Pathways to Nurse‐Community Partnerships for Climate Justice

While the Authentic Partnerships Model offers a framework for sustaining relationships, participants described processes that preceded formal collaboration. These findings were interpreted inductively and reflect the personal, ethical, and spiritual motivations that led PHNs and CBO representatives to seek partnership in the first place.

Participants shared how family histories, personal values, and direct encounters with environmental injustice shaped their journeys into partnership. They described a sense of calling or alignment between their professional work and the urgent needs of their communities. Some were drawn together through existing organizational connections, while others built new relationships from a shared recognition of the harms caused by climate injustice.

This theme explores how nurse‐community partnerships for climate justice often begin not with strategy but with a strong internal foundation and a desire to connect with others working toward collective transformation.

**2a. Having a strong internal foundation**. Before entering formal partnerships, many PHNs and CBO representatives described an inner alignment—a deeply personal sense of calling, responsibility, and moral clarity—that shaped their engagement in climate justice work. This internal foundation was built on family values, cultural teachings, personal experience, and an ethical drive to respond to injustice.

For PHNs, this foundation often emerged as a professional turning point or sense of misalignment with traditional healthcare roles. One PHN described how the pandemic catalyzed a career shift, “I don't want to be a school nurse anymore. This is not feeding my soul… I want to enter environmental health nursing. I want to do something where I can stand up and speak out for injustices.” [PHN8]. CBO partners similarly rooted their activism in familial relationships and community care. One shared,

“My grandmother always said that you don't go get an education or do better for yourself and forget where you come from, right? And because I was raising my kids in the same community, I wanted‐ I had a self‐interest that I wanted the community to benefit my family as I can benefit it… making sure that they will be safe, making sure that they were aware of the environmental hazards and harms that existed in poverty‐stricken communities.” [CBO4]

Alongside this values‐based orientation, participants emphasized the importance of building knowledge about environmental hazards affecting their communities. Learning was often self‐directed and relational, initiated by community meetings, informal networks, or chance encounters with environmental experts. One PHN described attending a local event out of curiosity and leaving transformed, “They want to put [an ethane cracker plant] in my neck of the woods… I didn't even know how plastic was made, but learned that day.” [PHN2]. A CBO reflected on a pivotal moment of understanding water pollution's health effects: *“*That was a huge moment—now I was able to go into human health and the injustice.” [CBO1]. Some PHNs reflected on global experiences with Indigenous communities that shifted their understanding of justice. One shared, “Why is it their responsibility to protect the environment when it's being globally degraded? Their voice was more important on what the reality was.” [PHN5] Participants also described a relational shift—broadening their concept of community beyond humans to include non‐human beings and ecosystems. One participant explained: “It really only sort of flips into thinking about working deeply with the community as a whole when you start to argue that climate justice is more than just working with humans; it's also working with non‐humans.” [PHN1].

This subtheme was interpreted inductively and expands the literature by revealing that nurse‐community partnerships are not just formed through shared goals—they are rooted in personal reckoning, deep learning, and an evolving recognition of interconnection. This foundation shaped how participants sought collaborations and positioned themselves in solidarity with communities facing environmental harm.

**2b. Seeking connection with others**. Once grounded in a sense of purpose and with a deeper awareness of environmental injustice, participants described actively seeking connections with others engaged in climate justice work. These connections often formed the initial pathways into nurse‐community partnerships through intentional outreach, shared networks, or, as participants sometimes described, “serendipity” or even spiritual alignment.

Some partnerships developed from pre‐existing relationships, such as friendships, academic collaborations, or prior work within grassroots organizations. Others were formed through fellowships, referrals, or organizational introductions. One PHN reflected on finding a role that aligned with her values through a networked opportunity, “I feel like the community found me… I feel like the universe brought me into [UNIVERSITY] to work with [DOCTOR] and the [NATIVE NATION].” [PHN8].

In some cases, partnerships were structurally facilitated. For example, several PHNs were matched with CBOs through a national nursing organization: “They had a partnership already… so it was like this partnership had already developed for me.” [PHN7]. Even when formal structures were in place, participants emphasized the importance of relational work in fostering trust and alignment. Participants shared stories of following their curiosity, listening deeply to community members, and leaning into moments of relational invitation or shared urgency.

Participants also noted how academic institutions, professional associations, and community networks could link PHNs and CBOs with shared visions and complementary skills. Professors, mentors, and organizational leaders often helped broker introductions, fostering connections that aligned around a common commitment to community health and environmental justice.

This subtheme was inductively derived and emphasizes that partnerships do not emerge from values alone—they require intentional relationship‐seeking**, **navigating systems, and being open to moments of mutual recognition. The findings underscore that connection is both a strategy and a relational process—one rooted in shared values and shaped by opportunities, introductions, and a readiness to collaborate.

These findings offer a nuanced account of how nurse‐community partnerships for climate justice are formed, sustained, and shaped by shared values, power‐sharing practices, and relational commitments. While many sustaining processes aligned with the Authentic Partnerships Model, participants also revealed pathways into partnership that extend beyond the model, grounded in personal transformation, deep listening, and a sense of interconnection with human and more‐than‐human communities. These insights lay the groundwork for interpreting how PHNs and CBOs navigate complexity, tension, and transformation in their collaborative climate justice efforts.

## 4 Discussion

This study explored how PHNs and CBOs form and sustain partnerships to promote climate justice, including their partnership processes, perceived facilitators and barriers, and the values that shape their relational work. While the Authentic Partnerships Model provided a valuable lens for understanding many of the relationship‐centered processes described by participants (CCPH Board of Directors 2013), the findings also extend beyond the model, highlighting the personal, ethical, and ecological foundations that shape how such partnerships begin and evolve.

Participants described their partnerships as deeply relational processes grounded in mutual trust, shared values, and reciprocal capacity‐building. These findings align with foundational scholarship on power‐sharing in public health collaborations (Heller et al. 2023) and relational public health nursing (Kulbok et al. 2012; Schaffer et al. 2022). Trust and reciprocity were not viewed as preliminary steps but as ongoing practices that sustain long‐term engagement. The emphasis on care, grief, and relational accountability aligns with identification of essential practices in power‐building health partnerships, where emotional and structural dimensions are interwoven to support transformational change (Gaydos et al. 2022). While this study focuses on climate justice, the relational processes and partnership values described by participants are consistent with public health nursing practices in other areas, including community resilience (Duva et al. 2022; Salt et al. 2020; Williams et al. 2018). These parallels suggest that climate justice partnerships can build on well‐established PHN strengths in community trust‐building, collaborative intervention design, and sustained engagement.

In addition to aligning with established principles of authentic partnership, participants’ narratives revealed that climate justice partnerships often begin with a personal reckoning, through which PHNs and CBO representatives deepen their awareness of environmental injustice, develop a sense of collective responsibility, and seek connection with others committed to community well‐being. These motivations reflect an “internal foundation” of values, purpose, and ecological interconnection consistent with the Planetary Health Education Framework (Faerron Guzmán et al. 2021), which calls on health professionals to recognize their relational place within a living world. Such foundations are also embedded in the revised Code of Ethics for Nurses, which states nurses have an obligation to engage in collective action to confront structural and environmental injustice (ANA 2025). The findings directly align with the research question by illuminating the relational processes that sustain nurse‐community partnerships and the internal motivations and values that give rise to them. Through deductive and inductive analysis, this study reveals that climate justice partnerships are shaped not only by formal principles of collaboration but also by transformative experiences rooted in family, culture, grief, land, and hope. These experiences resonate with prior work highlighting the role of emotional connection and cultural humility in PHN practice (Kerr et al. 2021; Cantu et al. 2016) and further underscore the importance of viewing partnership work as an ethical, rather than merely technical, undertaking. These findings align with existing PHN partnership models emphasizing community‐defined goals, shared accountability, and long‐term engagement (Anderson et al. 2002; Salt et al. 2020). They also reinforce the view that PHN skills such as relational communication, reflexivity, and collaboration are not only critical to health promotion broadly (Schaffer et al. 2022; Shigematsu et al. 2015) but essential for building capacity in climate justice efforts. Prior literature has called for more authentic community engagement in nursing and environmental health, yet few have detailed how nurses and grassroots leaders navigate partnerships in practice (LeClair, Watts, et al. 2021; Lilienfeld et al. 2018; Polivka and Chaudry 2018). This study begins to address that gap by offering grounded, narrative‐rich accounts of how trust is built, power is shared, and commitment is sustained. It contributes to a growing body of work that positions PHNs as active practitioners and collaborators in justice‐driven community work (Kerr et al. 2021; LeClair, Dudek, et al. 2024; LeClair et al. 2022; LeClair, Kunkul, et al. 2024; Lilienfeld et al. 2018). While participants described deeply relational and values‐based partnerships, they also identified barriers that complicated their ability to fully enact shared principles. The geographic distance between PHNs and CBOs created logistical and relational challenges, limiting the PHNs’ ability to connect partners with local resources. Several PHNs noted that being based in academic institutions sometimes limited their advocacy ability, particularly around controversial or politically sensitive issues, such as worker protections or environmental regulation. These findings mirror broader critiques of how bureaucratic structures and risk‐averse institutions can limit equitable collaboration and suppress policy engagement (Aivalli et al. 2025; Luthuli et al. 2024). They point to the need for more flexible systems that enable PHNs to engage in policy change alongside communities, especially in the face of climate‐related health inequities. This study affirms that sustaining nurse‐community partnerships for climate justice requires more than tools or frameworks. It requires a commitment to relational ways of knowing and being, rooted in mutual trust, accountability, and shared purpose. These findings underscore that climate justice is not only an outcome but a process that unfolds through relationships built on care, humility, and hope. These relational processes are not only essential for trust‐building and sustained collaboration but also serve as the foundation for impactful climate justice strategies. For example, nurse–CBO partnerships have led to interventions such as air and soil quality monitoring, food sovereignty, and policy advocacy focused on environmental protections and labor rights. These strategies and their outcomes are described in companion papers focused on climate justice strategies (LeClair, Dudek, et al. 2024) and outcomes (LeClair et al. under review). Together, these works illustrate how strong relational foundations enable PHNs to engage in climate justice efforts that produce tangible environmental and health equity benefits.

### 4.1 Limitations

Volunteer and academic PHNs collaborated with CBO representatives to address climate justice in six states. Consequently, the findings may not apply to other geographic areas or settings where PHNs work, such as local, state, and tribal health departments. While many participants openly shared their positionalities, such as race and gender, they were not directly prompted to provide demographic information, resulting in our inability to consider these factors in the analysis and interpretation of the study's findings.

## 5 Recommendations for Public Health Nursing

Public health nurses must be supported in developing and sustaining authentic partnerships that address climate justice through relational, community‐based practices. These findings align with prior calls to reimagine nursing roles through a justice and systems lens (LeClair et al. 2022; Nicholas and Breakey 2017). Support should include reflective education, institutional flexibility, and public health systems that value long‐term, equity‐driven engagement (Gaydos et al. 2022; Schaffer et al. 2022). Creating space for advocacy, relationship‐building, and values‐based practice is essential for PHNs to act as effective partners in climate justice efforts.

### 5.1 Practice

These findings suggest that PHNs engaged in climate justice must be prepared to act as relationship‐builders, advocates, and cultural learners, not just public health professionals. Similar to insights from community‐based research and government partnerships (Andress et al. 2020; Heller et al. 2023; Simon‐Ortiz et al. 2024), successful collaboration requires communication, power‐sharing, and reflexivity. The Public Health Intervention Wheel identifies collaboration as a core nursing intervention (Schaffer et al. 2022), and this study reinforces that collaboration is not simply a task but a relational process that requires sustained commitment and responsiveness to community‐defined priorities. Public health systems must create the conditions for PHNs to do this work, including time, flexibility, and meaningful integration with community structures (Bekemeier et al. 2025).

### 5.2 Education

Educators can play a critical role in preparing nurses for climate justice partnerships by helping them cultivate the internal foundations described by participants, including clarity of values, humility, and understanding how environmental injustice contributes to health inequities. The Code of Ethics for Nurses (American Nurses Association 2025) and the AACN Essentials (AACN 2021) already provide guidance on integrating planetary health and social justice into nursing curricula. Building on the Planetary Health Education Framework (Faerron Guzmán et al. 2021), this study affirms that integration must extend beyond content delivery to include experiential and practice‐based learning, reflective pedagogy, and authentic engagement with communities most impacted by environmental harm (Evans‐Agnew et al. 2024). Curricula should also incorporate collaboration and advocacy skills to equip nurses to engage in ethical and relational partnership work (LeClair, Dudek, et al. 2024).

### 5.3 Research

Future research should investigate how PHNs form and sustain partnerships across public health settings, including local and tribal health departments (Bekemeier et al. 2025). Comparative studies could illuminate how institutional context and positionality shape relational work and advocacy capacity (Aivalli et al. 2025). Longitudinal research is also needed to examine partnership evolution and outcomes, including how alliances adapt or dissolve in response to shifting political and ecological contexts. Researchers may consider adapting frameworks such as the Authentic Partnerships Model (CCPH 2013) or developing new nursing‐led partnership theories that honor the relational and justice‐oriented nature of this work. Several scholars note the importance of power awareness in partnership frameworks, suggesting the need for methods and measures that explicitly examine equity and transformation within nurse‐community partnerships (Andress et al. 2020; Oetzel et al. 2022).

## 6 Conclusions

This study describes how PHNs and CBO representatives collaborate to advance climate justice. Findings reveal that successful partnerships are grounded in trust, care, mutual respect, and shared purpose, but also shaped by institutional constraints, geographic distance, and the need for ongoing negotiation. Beyond documenting partnership processes, this study illuminates the personal and collective values that initiate and sustain justice‐driven collaboration.

By identifying the processes that sustain partnerships and the pathways through which they form, this research expands current models of authentic collaboration. It offers guidance for PHNs, educators, and public health systems. Supporting PHNs in this work requires skills training and structural and cultural shifts that honor community leadership, enable advocacy, and prioritize long‐term relationship building. As climate injustices intensify, PHNs have a critical role as partners committed to co‐creating health and justice with the communities they serve.

## Funding

Funding was received for this study from the Midwest Nursing Research Society, Sigma Theta Tau International, the University of Wisconsin–Madison School of Nursing, the University of Wisconsin‐Madison Global Health Institute, and the University of Wisconsin‐Madison Nelson Institute for Environmental Studies.

## Conflicts of Interest

The authors declare no conflicts of interest.

## Data Availability

The data are not publicly available due to privacy or ethical restrictions.

## References

[phn70044-bib-0001] AACN . 2021. The Essentials: Core Competencies for Professional Nursing Education. American Association of Colleges of Nursing. www.aacnnursing.org/Portals/42/AcademicNursing/pdf/Essentials‐2021.pdf. 10.1016/j.profnurs.2025.02.003 40074377

[phn70044-bib-0002] Aivalli, P. , S. Dada , B. Gilmore , P. N. Srinivas , and A. De Brún . 2025. “Power Dynamics and Intersectoral Collaboration for Health in Low‐and Middle‐Income Countries: A Realist Review.” Health Policy and Planning 40, no. 6: 661–683. 40186364 10.1093/heapol/czaf022 PMC12160828

[phn70044-bib-0003] American Nurses Association . 2025. Code of Ethics for Nurses. American Nurses Association.

[phn70044-bib-0004] American Nurses Association . 2022. Public Health Nursing: Scope and Standards of Practice. 3rd ed. American Nurses Association.

[phn70044-bib-0005] Amiri, A. , and S. Zhao . 2019. “Working With an Environmental Justice Community: Nurse Observation, Assessment, and Intervention.” Nursing Forum 54, no. 2: 270–279. 10.1111/nuf.12327. 30690745

[phn70044-bib-0006] Anderson, D. , T. Guthrie , and R. Schirle . 2002. “A Nursing Model of Community Organization for Change.” Public Health Nursing 19, no. 1: 40–46. 11841681 10.1046/j.1525-1446.2002.19006.x

[phn70044-bib-0007] Andress, L. , T. Hall , S. Davis , J. Levine , K. Cripps , and D. Guinn . 2020. “Addressing Power Dynamics in Community‐Engaged Research Partnerships.” Journal of Patient‐Reported Outcomes 4, no. 1: 24. 32249348 10.1186/s41687-020-00191-z PMC7131972

[phn70044-bib-0008] Baptista, A. I. , S. Jesudason , M. Greenberg , and A. Perovich . 2023. “Landscape Assessment of the US Environmental Justice Movement: Transformative Strategies for Climate Justice.” Environmental Justice 16, no. 2: 1–7. 10.1089/env.2021.0075.

[phn70044-bib-0009] Bekemeier, B. , P. M. Kett , G. Whitman , K. Chadwick , and J. K. Edmonds . 2025. “Distribution and Specialties of Broadly Versus Narrowly Defined Public Health Nurses Working in Government Settings in the United States, 2022.” American Journal of Public Health 115, no. 4: 536–545. 39946677 10.2105/AJPH.2024.307950 PMC11903085

[phn70044-bib-0010] Benz, S. A. , and J. A. Burney . 2021. “Widespread Race and Class Disparities in Surface Urban Heat Extremes Across the United States.” Earth's Future 9, no. 7: e2021EF002016.

[phn70044-bib-0011] Boeije, H. 2002. “A Purposeful Approach to the Constant Comparative Method in the Analysis of Qualitative Interviews.” Quality and Quantity 36: 391–409. 10.1023/A:1020909529486.

[phn70044-bib-0012] Braun, V. , and V. Clarke . 2006. “Using Thematic Analysis in Psychology.” Qualitative Research in Psychology 3: 77–101. 10.1191/1478088706qp063oa.

[phn70044-bib-0013] Braun, V. , and V. Clarke . 2019. “Reflecting on Reflexive Thematic Analysis.” Qualitative Research in Sport, Exercise and Health 11, no. 4: 589–597. 10.1080/2159676X.2019.1628806.

[phn70044-bib-0014] Bullard, R. 1994. “The Legacy of American Apartheid and Environmental Racism.” Journal of Civil Rights and Economic Development 9, no. 2: 445–474.

[phn70044-bib-0015] Buse, C. G. , and R. Patrick . 2020. “Climate Change Glossary for Public Health Practice: From Vulnerability to Climate Justice.” Journal of Epidemiology and Community Health 74: 867–871. 10.1136/jech-2020-213889. 32620579

[phn70044-bib-0016] Cantu, A. , M. A. Graham , A. V. Millard , et al. 2016. “Environmental Justice and Community‐Based Research in Texas Borderland Colonias.” Public Health Nursing 33, no. 1: 65–72. 10.1111/phn.12187. 25787846 PMC4575224

[phn70044-bib-0017] CCPH Board of Directors . 2013. “Position Statement on Authentic Partnerships.” Community‐Campus Partnerships for Health. https://ccphealth.org/partnering/principles‐of‐partnering/.

[phn70044-bib-0018] DeSalvo, K. B. , Y. C. Wang , A. Harris , J. Auerbach , D. Koo , and P. O'Carroll . 2017. “Peer Reviewed: Public Health 3.0: A Call to Action for Public Health to Meet the Challenges of the 21st Century.” Preventing Chronic Disease 14: E78. 28880837 10.5888/pcd14.170017 PMC5590510

[phn70044-bib-0019] Duva, I. M. , J. R. Murphy , and L. Grabbe . 2022. “A Nurse‐Led, Well‐Being Promotion Using the Community Resiliency Model, Atlanta, 2020–2021.” American Journal of Public Health 112, no. S3: S271–S274. 35679550 10.2105/AJPH.2022.306821 PMC9184890

[phn70044-bib-0020] Enlow, P. T. , C. Thomas , A. M. Osorio , et al. 2024. “Community Partnership to Co‐Develop an Intervention to Promote Equitable Uptake of the COVID‐19 Vaccine Among Pediatric Populations.” Delaware Journal of Public Health 10, no. 1: 30. 10.32481/djph.2024.03.06 PMC10987021 38572140

[phn70044-bib-0021] Evans‐Agnew, R. , J. LeClair , and D. Sheppard . 2024. “Just‐Relations and Responsibility for Planetary Health: The Global Nurse Agenda for Climate Justice.” Nursing Inquiry 31, no. 1: e12563. 37256546 10.1111/nin.12563

[phn70044-bib-0022] Faerron Guzmán, C. A. , A. A. Aguirre , B. Astle , et al. 2021. “A Framework to Guide Planetary Health Education.” Lancet Planetary Health 5, no. 5: e253–e255. 10.1016/S2542-5196(21)00110-8. 33894134

[phn70044-bib-0023] Forman, F. , G. Solomon , R. Morello‐Frosch , and K. Pezzoli . 2016. “Chapter 8. Bending the Curve and Closing the Gap: Climate Justice and Public Health.” Collabra 2, no. 1: 1–17. 10.1525/collabra.67.

[phn70044-bib-0024] Gaydos, M. , V. Do‐Reynoso , M. Williams , H. Davalos , and A. J. López . 2022. “Power‐Building Partnerships for Health: Lessons From Santa Barbara About Building Power to Protect Farmworker Health and Advance Health Equity.” Journal of Public Health Management and Practice 28, no. 4: S166–S170. 35616562 10.1097/PHH.0000000000001485

[phn70044-bib-0025] Golden, T. L. , and M. L. Wendel . 2020. “Public Health's Next Step in Advancing Equity: Re‐Evaluating Epistemological Assumptions to Move Social Determinants From Theory to Practice.” Frontiers in Public Health 8, no. 131: 1–7. 10.3389/fpubh.2020.00131. 32457863 PMC7221057

[phn70044-bib-0026] Hayden, M. H. , P. J. Schramm , C. B. Beard , et al. 2023. “Human health.” In Fifth National Climate Assessment, edited by A. R. Crimmins , C. W. Avery , D. R. Easterling , K. E. Kunkel , B. C. Stewart , and T. K. Maycock . Global Change Research Program. 10.7930/NCA5.2023.CH15.

[phn70044-bib-0027] Heller, J. C. , O. M. Little , V. Faust , et al. 2023. “Theory in Action: Public Health and Community Power Building for Health Equity.” Journal of Public Health Management and Practice 29, no. 1: 33–38. 36448756 10.1097/PHH.0000000000001681

[phn70044-bib-0028] Kang, R. 1995. “Building Community Capacity for Health Promotion: A Challenge for Public Health Nurses.” Public Health Nursing 12, no. 5: 312–318. 7479539 10.1111/j.1525-1446.1995.tb00154.x

[phn70044-bib-0029] Kerr, R. , C. Cook , N. Chaney , M. Sotor , and K. Huffling . 2021. “Nurses Heal Environmental Injustice Through Community Partnerships.” Environmental Justice 15, no. 2: 90–97. 10.1089/env.2021.0041.

[phn70044-bib-0030] Kuehnert, P. , J. Fawcett , K. DePriest , et al. 2022. “Defining the Social Determinants of Health for Nursing Action to Achieve Health Equity: A Consensus Paper From the American Academy of Nursing.” Nursing Outlook 70, no. 1: 10–27. 10.1016/j.outlook.2021.08.003. 34629190

[phn70044-bib-0031] Kulbok, P. A. , E. Thatcher , E. Park , and P. S. Meszaros . 2012. “Evolving Public Health Nursing Roles: Focus on Community Participatory Health Promotion and Prevention.” Online Journal of Issues in Nursing 17, no. 2: 1. 22686109

[phn70044-bib-0032] LeClair, J. , A. Dudek , and S. Zahner . 2024. “Climate Justice Strategies Implemented by Public Health Nurses and Their Community Partners.” Journal of Advanced Nursing 0: 1–15. 10.1111/jan.16598. PMC12623680 39526565

[phn70044-bib-0033] LeClair, J. , A. Dudek , and S. Zahner . 2025. “Climate Justice Perspectives and Experiences of Nurses and Their Community Partners.” Nursing Inquiry 32, no. 1: e12690. 39679844 10.1111/nin.12690 PMC11648356

[phn70044-bib-0034] LeClair, J. , R. Evans‐Agnew , and C. Cook . 2022. “Defining Climate Justice in Nursing for Public and Planetary Health.” American Journal of Public Health 112, no. 55: S256–S258. 10.2105/AJPH.2022.306867. 35679549 PMC9184902

[phn70044-bib-0035] LeClair, J. , F. Kunkul , H. Olson‐Williams , et al. 2024. “Public Health Nurse Partnerships to Advance Environmental Justice and Heart Health in a Black Community.” Environmental Justice 0: 1–8. 10.1089/env.2024.0023.

[phn70044-bib-0036] LeClair, J. , J. Luebke , and L. D. Oakley . 2025. “Critical Environmental Justice Nursing for Planetary Health: A Guiding Framework.” Advances in Nursing Science 48, no. 4: 345–354. 10.2105/AJPH.2022.306867. 34569987

[phn70044-bib-0037] LeClair, J. , T. Watts , and S. Zahner . 2021. “Nursing Strategies for Environmental Justice: A Scoping Review.” Public Health Nursing 38, no. 2: 296–308. 10.1111/phn.12840. 33210747

[phn70044-bib-0038] Lemon, S. C. , H. A. Joseph , S. Williams , et al. 2023. “Reimagining the Role of Health Departments and Their Partners in Addressing Climate Change: Revising the Building Resilience Against Climate Effects (BRACE) Framework.” International Journal of Environmental Research and Public Health 20, no. 15: 6447. 37568988 10.3390/ijerph20156447 PMC10419192

[phn70044-bib-0039] Lilienfeld, E. , P. K. Nicholas , S. Breakey , and I. B. Corless . 2018. “Addressing Climate Change Through a Nursing Lens Within the Framework of the United Nations Sustainable Development Goals.” Nursing Outlook 66, no. 5: 482–494. 10.1016/j.outlook.2018.06.010. 30172574

[phn70044-bib-0040] Luthuli, S. , M. Daniel , and J. H. Corbin . 2024. “Power Imbalances and Equity in the Day‐to‐Day Functioning of a North Plus Multi‐South Higher Education Institutions Partnership: A Case Study.” International Journal for Equity in Health 23, no. 1: 59. 38491440 10.1186/s12939-024-02139-x PMC10943907

[phn70044-bib-0054] LeClair, L. , A. Van Aartsen , and B. Bekemeier . Public health nurse-community partnership outcomes for climate justice. (under review).

[phn70044-bib-0041] National Community‐Based Organization Network . 2004. “What Is a CBO?” National Community‐Based Organization Network. https://sph.umich.edu/ncbon/about/whatis.html.

[phn70044-bib-0042] Nicholas, P. K. , and S. Breakey . 2017. “Climate Change, Climate Justice, and Environmental Health: Implications for the Nursing Profession.” Journal of Nursing Scholarship 49, no. 6: 606–616. 10.1111/jnu.12326. 28749596

[phn70044-bib-0043] Oetzel, J. G. , B. Boursaw , M. Magarati , et al. 2022. “Exploring Theoretical Mechanisms of Community‐Engaged Research: A Multilevel Cross‐Sectional National Study of Structural and Relational Practices in Community‐Academic Partnerships.” International Journal for Equity in Health 21, no. 1: 59. 35501798 10.1186/s12939-022-01663-y PMC9063068

[phn70044-bib-0044] Polivka, B. J. , and R. V. Chaudry . 2018. “A Scoping Review of Environmental Health Nursing Research.” Public Health Nursing 35, no. 1: 10–17. 10.1111/phn.12373. 29164725

[phn70044-bib-0045] Postma, J. 2008. “Elucidating Empowerment in El Proyecto Bienestar (the Well‐Being Project).” Journal of Advanced Nursing 62, no. 4: 441–450. 18476944 10.1111/j.1365-2648.2008.04605.x

[phn70044-bib-0046] Rockstrom, J. , J. Gupta , D. Qin , S. Lade , J. Abrams , and L. Andersen . 2023. “Safe and Just Earth System Boundaries.” Nature 619: 102–111. 10.1038/s41586-023-06083-8. 37258676 PMC10322705

[phn70044-bib-0047] Rubin, H. , and I. Rubin . 2011. “Choosing Interviewees and Judging What They Say.” In Qualitative Interviewing: The Art of Hearing Data, 65–92. Sage.

[phn70044-bib-0048] Salt, R. J. , C. Sickora , T. L. Page , et al. 2020. ““We Didn't Forget” Utilizing a Community‐Nurse Partnership to Promote Health in Rockport, Texas After Hurricane Harvey.” Public Health Nursing 37, no. 1: 113–120. 31713275 10.1111/phn.12684

[phn70044-bib-0049] Schaffer, M. , S. Strohschein , and K. Glavin . 2022. “Twenty Years With the Public Health Intervention Wheel: Evidence for Practice.” Public Health Nursing 39: 195–201. 10.1111/phn.12941. 34231267

[phn70044-bib-0050] Shigematsu, Y. , Y. Hatano , and H. Kimura . 2015. “A Partnership Development Process Assessment Scale for Public Health Nurses in Japan.” Public Health Nursing 32, no. 3: 266–276. 25731806 10.1111/phn.12178

[phn70044-bib-0051] Simon‐Ortiz, S. , S. Bilick , M. Frey , et al. 2024. “Community Power–Building Groups and Public Health NGOs: Reimagining Public Health Advocacy.” Health Affairs 43, no. 6: 798–804. 10.1377/hlthaff.2024.00035. 38830166

[phn70044-bib-0052] Wang, C. , and M. A. Burris . 1997. “Photovoice: Concept, Methodology, and Use for Participatory Needs Assessment.” Health Education & Behavior 24, no. 3: 369–387. 9158980 10.1177/109019819702400309

[phn70044-bib-0053] Williams, M. V. , A. Chandra , A. Spears , et al. 2018. “Evaluating Community Partnerships Addressing Community Resilience in Los Angeles, California.” International Journal of Environmental Research and Public Health 15, no. 4: 610. 29584681 10.3390/ijerph15040610 PMC5923652

